# How Inhibitory Control Relates to Positive and Negative Affective States in Red Junglefowl

**DOI:** 10.3389/fvets.2022.872487

**Published:** 2022-04-08

**Authors:** Laura Clare Garnham, Charlie Clarke, Hanne Løvlie

**Affiliations:** ^1^Division of Biology, Department of Physics, Chemistry and Biology, Linköping University, Linköping, Sweden; ^2^School of Biological Sciences, University of Manchester, Manchester, United Kingdom

**Keywords:** affective state, animal welfare, chicken, cognitive bias, fowl, impulsivity, tonic immobility

## Abstract

Individual differences in inhibitory control, an aspect of cognition, are found in many species. How this variation links to affective states is not much explored, and could be relevant for welfare. As less fearful, more optimistic, individuals may act more impulsively, inhibitory control could link to less negative, more positive, affective states. Alternatively, poorer inhibitory control could associate with more negative, less positive, affective states, as poorer inhibitory control can result in individuals being less able to adapt to changing environments and more likely to show stereotypies. We here explored in three cohorts (*N* = 209) of captive red junglefowl, the ancestor of domestic chickens, how inhibitory control associated with affective states. Specifically, we measured inhibitory control with a detour task, and negative and positive affective states with a tonic immobility test and a cognitive judgement bias test, respectively. Cognition and behaviour can differ between ages and sexes. Therefore, we investigated how inhibitory control related to affective states in younger chicks (≈2.5 weeks old), older chicks (≈5 weeks old) and sexually mature adults (≈28 weeks old) of both sexes. In younger chicks, poorer inhibitory control associated with less negative, more positive, affective states. We found no relationship between inhibitory control and affective states in older chicks or adults, nor sex differences regarding how inhibitory control related to affective states. Overall, our results suggest that inhibitory control can link to affective states and that the nature of these links can change over ontogeny.

## Introduction

Individual variation in cognition [i.e., how individuals acquire, process, store, and act on environmental information (1)] is repeatedly observed across taxa [e.g., (2–4)]. Nevertheless, the implications of this variation for individuals are not well-known. Determining how cognition links to affective states could help us ensure good welfare for animals. Affective states (i.e., emotional states) can be perceived as negative [e.g., stress (5), fear (6, 7)], or positive [e.g., pleasure (8)]. In turn, more positive and less negative affective states could be considered an indicator of good welfare (9, 10). Thus, it is not surprising that how cognition and affective states relate has become a focus of recent research. Thus far, affective state has been found to influence various aspects of individual variation in cognition, including learning (11) and decision making (12). Nonetheless, there are still potential relationships between cognition and affective states that are not much explored.

One potential relationship between cognition and affective states that is scarcely investigated is how inhibitory control relates to affective states. Inhibitory control [an executive function which allows individuals to inhibit their prepotent responses (13)] is an aspect of cognition repeatedly shown to vary between individuals of the same species [e.g., (14–16)]. Inhibitory control can be measured using a detour task, which explores whether an individual can inhibit the prepotent response of trying to obtain a reward directly through a transparent barrier and instead obtain it by detouring around the barrier [e.g., (14, 17, 18)]. A potential functional cause for a relationship between inhibitory control and affective states in non-human animals could be neuropsychology, specifically, how risk-seeking vs. avoidant individuals are. Individuals with less negative, and more positive, affective states are likely to be less fearful [e.g., (6, 7)] and more optimistic [e.g., (19, 20)]. A lower fearfulness implies that these individuals will be less concerned about potential risks, while higher optimism implies that they are more likely to assume that taking risks will result in positive consequences. On the other hand, individuals with more negative and less positive affective states could be expected to be more fearful [e.g., (6, 7)] and less optimistic [e.g., (19, 20)], which could lead to these individuals being more avoidant. Taken together, we could expect individuals with more risk-seeking, impulsive behaviour (i.e., showing poorer inhibitory control) to show more positive, less negative, affective states. To our knowledge, this expectation has not yet been empirically explored. Inhibitory control could also connect to affective state via the consequences that poorer inhibitory control can have for individuals. First, individuals with poorer inhibitory control are less able to adapt to changing situations [e.g., (21)]. Second, individuals with poorer inhibitory control are generally more impulsive [e.g., (22–24)], which can make them more likely to display behaviours with seemingly negative consequences [e.g., stereotypies (25–27)]. Taken together, poorer inhibitory control could be predicted to have negative consequences for individuals, and thus link to more negative, less positive, affective states. That aspects of inhibitory control can be worsened by stress-inducing experiences [e.g., cortisol dosing (28), forced isolation (29)] supports such a link, though further studies are needed to confirm or reject this.

To determine how variation in inhibitory control could influence affective states, we need to be able to measure affective states. Animals with more negative affective states [e.g., more fearful (6, 7), more stressed (30, 31)] remain longer in tonic immobility, that is they remain motionless for longer after having been restrained on their back and tonic immobility has been induced (32). Animals with more positive affective states show higher levels of positive judgement bias [i.e., optimism (e.g., 19, 20)]. More optimistic individuals are faster to approach a novel ambiguous cue that is intermediate between learnt positive and negative cues in a cognitive judgement bias test [e.g., (33, 34)]. If poorer inhibitory control links to more negative, less positive, affective states, it should correlate positively with negative affective states and negatively with positive affective states. Impulsivity (thus also inhibitory control) can link to affective states in humans. For example, in humans, higher impulsivity regarding alcohol consumption has been found to link to both more negative and more positive affective states (35), and increased impulsivity is associated with increases in daily stress (36). Overall, how impulsivity relates to affective state in humans appears to depend on the measures of impulsivity and affective states explored [reviewed in (37)]. Despite the interest in how inhibitory control connects to affective states in humans, this connection is yet to be investigated in other animals.

If inhibitory control links to affective states there is potential for these relationships to differ over ontogeny (i.e., different relationships may be found at different ages). For example, inhibitory control can be slow to develop {at least in some primates, [e.g., humans and rhesus monkeys, *Macaca mulatta*, (38, 39)]}. Further, individuals can learn to improve their inhibitory control over time (18, 40). Taken together, this suggests that individuals should improve in inhibitory control as they age. This has been observed in humans [e.g., (41, 42)], but lacks research in other animals. Less is known about changes in affective states over ontogeny, however that inhibitory control can change over ontogeny implies that the relationship between inhibitory control and affective states may also do so. Relationships between inhibitory control and affective states could also differ between sexes. Both inhibitory control and affective states show sex differences. Males typically have poorer inhibitory control (when it comes to avoiding impulsive actions), but are better at inhibiting impulsive choices (i.e., waiting for a larger, delayed reward rather than going for an instant, small reward), than females (43). The nature of sex differences in affective states differs between species (44). Overall, while sex differences in the relationship between inhibitory control and affective states could be expected, they are less clear to predict the nature of.

We here explored how inhibitory control relates to affective states in red junglefowl, *Gallus gallus*. If inhibitory control is linked to affective states via how risk seeking vs. risk avoidant individuals are, we hypothesised that individuals with poorer inhibitory control would have a less negative, more positive, affective states. If inhibitory control linked to affective states due to poorer inhibitory control having negative consequences, we hypothesised that individuals with poorer inhibitory control would have more negative, less positive, affective states would have. Red junglefowl, along with their descendant, the domestic chicken (45), are increasingly used for behavioural and cognitive studies [reviewed in (46)]. Chickens are one of the world's most intensively farmed animals [reviewed by (47, 48)], and face severe welfare issues such as feather pecking, vent pecking, and cannibalism (27, 49). The population of junglefowl we used for this study are known to show individual variation in inhibitory control [e.g., (40, 50)], which can show temporal consistency across time in both chicks (51) and adults (52). Fowl are known to show easily discernable tonic immobility reactions (53), thus tonic immobility can be used to measure negative affective states in this species. Cognitive judgement bias tests are validated for measuring positive affective states or optimism in non-human animals (54, 55) and the cognitive judgement bias test used here was specifically developed for use in junglefowl (34, 50, 56). As junglefowl and domestic chickens develop from chicks to adults, they may display changes in behaviour and cognition, as well how these relate to each other [e.g., (34, 57–59)]. Thus, we explored relationships between inhibitory control and affective states in three ages, young chicks (≈2.5 weeks), older chicks (≈5 weeks) and sexually mature adults (≈28 weeks). We also included both males and females in this study to explore whether relationships between inhibitory control and affective states differed between sexes.

## Materials and Methods

### Animals and Housing

The red junglefowl used for this study came from a pedigree bred population belonging to Linköping University, Sweden [see (56) for further details]. Specifically, we used three cohorts (Cohort 1 was hatched in 2016, Cohort 2 in 2017, and Cohort 3 in 2019). Birds were tested between 1 and 6 weeks of age [i.e., as chicks, before the age at which they would typically become independent from their mothers, (60, 61)] and between 27 and 29 weeks of age [i.e., as sexually mature adults, sexual maturity occurs at around 20–25 weeks of age, (62, 63)]. We collected data from all cohorts when they were young chicks, ≈2.5 weeks old when their inhibitory control was measured (Cohort 1: *N*_females_ = 36, *N*_males_ = 34; Cohort 2: *N*_females_ = 23, *N*_males_ = 29; Cohort 3: *N*_females_ = 35, *N*_males_ = 23). We did not test all cohorts at all ages, rather we tested Cohort 1 also as adults, ≈28 weeks old when their inhibitory control was measured (*N*_females_ = 51, *N*_males_ = 48), and Cohort 3 also as older chicks, ≈5 weeks old when their inhibitory control was measured (*N*_females_ = 35, *N*_males_ = 23). Not all younger chicks tested in Cohort 1 were retested as adults (42 younger chicks from Cohort 1 were retested as adults), nor were all younger chicks tested in Cohort 3 retested as older chicks (54 younger chicks from Cohort 3 were retested as older chicks). Also, in Cohort 1, some of the birds that were tested as adults were not tested as younger chicks, which was due to that these birds had been control birds for other studies when they were chicks. Chicks were sexed at 6 weeks of age, when moulting into sex specific plumage. Thus, for both younger and older chicks, experimenters were blind to the chicks' sex. We used artificial incubators to hatch our birds, thus reducing potential maternal effects. We gave each bird a numbered wing tag soon after hatching to enable individual identification. As chicks, birds were housed in mixed-sex groups in cages (72 × 71 × 53 cm, L × W × H), which contained perches, heaters, and saw-dust for dustbathing. In 2016 and 2017, we distributed chicks evenly between the cages, whereas in 2019, we housed them either in small (consisting of seven individuals) or large (consisting of 16 individuals) groups as part of another study. In 2016 and 2019, we designated a home pen to each chick, so they lived in stable social groups, while in 2017, we regularly moved chicks between pens. Differences in how cohorts were kept, as chicks, were due to differences in other studies taking place in parallel. As adults, birds were kept in two single-sex enclosures (6 m^3^) equipped with perches, shelters, saw-dust for dustbathing, and access to an outdoor area (400 × 260 × 250 cm, L × W × H). For both chicks and adults, we used artificial lighting that was set so that the lights were on between 7 a.m. and 7 p.m., and birds always had access to *ad libitum* commercial poultry feed and water. Testing took place between 8 a.m. and 6 p.m. and birds were tested singly. The experiments were consistent with Swedish ethical requirements (Linköping Ethical Committee, ethical permit numbers 50-13 and 288-2019).

### Experimental Set-Up

Birds took part in tests in the following order: cognitive judgement bias test, detour task, tonic immobility test. Birds participated once in the tonic immobility and cognitive judgement bias tests (apart from in Cohort 1, where birds participated in these tests once as young chicks, and once as adults). Thus, we used the same measures of negative affective state and positive affective state for analyses of both younger chicks and older chicks. The detour task and cognitive judgement bias test took place in arenas which varied in their dimensions according to whether chicks or adults were being tested (dimensions of arena used for chicks: 48 × 39 × 15 cm; dimensions of arena used for adults: 90 × 50 × 60 cm, L × W × H). To minimise isolation stress during testing, we habituated all subjects, when they were chicks, to being alone in the testing arenas before they were tested [*sensu* (64)]. Rewards during testing and training always consisted of ≈1/3 of a fresh mealworm for chicks, and a whole fresh mealworm for adults. For tests that consisted of a training phase and a testing phase (i.e., detour task, cognitive judgement bias test), birds were returned to their cage for a minimum of 1 h after completing training before commencing testing, to maintain reward motivation and minimise duration of time spent in social isolation. Birds were sometimes initially helped during training to find rewards. This was done by either tapping near the reward with tweezers, leaving a trail of mealworms, or guiding with a hand. Birds were never helped to find rewards during testing. For tests that consisted of multiple trials (i.e., detour task, cognitive judgement bias test) a trial started after a bird was placed in the arena and ended either when the bird obtained the reward (both tests), approached a cue within 2 cm (cognitive judgement bias test, at this distance a bird can see if their chosen cue is rewarded or not), or left the arena (both tests). At the start of trials, in tests which involved interacting with testing equipment (i.e., detour task, cognitive judgement bias test), for both training and testing trials, birds were placed into the arena at one of the short ends of the arena opposite, and facing away, from the testing equipment. This latter prevented them from automatically approaching the testing equipment without intending to. Testing equipment was repositioned and rebaited between trials, without birds being able to watch this.

### Measuring Inhibitory Control

To measure inhibitory control, we used a detour task [*sensu* (40, 50)]. For each age, each bird was only tested once. Inhibitory control shows moderate temporal consistency between younger and older chicks in this population [Cohort 3 used in this study, (51)], and in adults (52).

#### Training Phase

Before a bird could take part in the detour task, we needed to familiarise it with obtaining a reward by navigating around a barrier (18). Specifically, birds learnt, over a series of trials, to obtain a reward from the centre of an opaque tube (5Ø × 8L cm for chicks and 7Ø × 8L cm for adults) by walking to one of the ends of the tube and putting their head into one of the tube's openings. We considered a bird ready for testing once it had obtained the reward, without pecking at the tube or needing help to find the reward, in five consecutive trials.

#### Testing Phase

During the testing phase of the detour task, we presented each bird with a transparent tube with a reward at its centre. The dimensions of the tube, as well as the position of both the bird and tube, at the start of each trial, were identical to those in the training phase. We measured each bird's inhibitory control as the number of trials (out of five) in which it inhibited the impulsive response of trying to peck the reward directly through the transparent tube, and instead used the detour learnt in the training phase to obtain the reward. We termed this measure “Inhibitory control,” where a higher measure indicated better inhibitory control (14). We used only five trials to reduce aspects of learning affecting this measure (18, 40). Variation in inhibitory control measures ranged from 0 to 5 for all ages.

### Measuring Negative Affective State

To measure negative affective state, we used a tonic immobility test [e.g., (7, 65), *sensu* (50, 57)]. Tonic immobility has shown moderate temporal consistency in our population of junglefowl for both chicks (58) and adults (57). We induced tonic immobility by laying a bird on its back in a V-shaped wooden cradle (20 × 10 cm) and gently holding the bird down, for 15 s, with one hand over its chest, applying light pressure, and another over its eyes. After this, we slowly removed our hands and measured “Negative affective state” as the time taken (s) by the bird to return to standing; the longer this latency, the more negative [i.e., more fearful (6, 7), more stressed (30, 31)] the affective state of the bird [e.g., (65, 66)]. While testing, the experimenter avoided eye contact with the test bird. If, following restraint, the bird did not remain on its back for at least 3 s, we did not consider tonic immobility to be induced, so we repeated the restraint. In 2016 and 2017, we used a maximum of three attempts to induce tonic immobility, while, in 2019, this was increased to 5. If we were unable to induce tonic immobility in a bird, we gave it a “Negative affective state” measure of 0 s. If a bird remained immobile for 600 s, we gave it a “Negative affective state” measure of 600 s and then gently brought it out of tonic immobility by hand. Eight younger chicks, three older chicks and in 15 adults were given a measure of 600 s for “Negative affective state.” Four younger chicks, and three adults, were not tested in the tonic immobility test. This was due to these birds being accidentally omitted from data collection due to experimenter error. Variation in our negative affective state measure ranged from 0 to 600 s for chicks and 4.37–600 s for adults.

### Measuring Positive Affective State

To measure positive affective state, we used a cognitive judgement bias test [*sensu* (34)].

#### Training Phase

Before a bird could participate in our cognitive judgement bias test, it needed to learn to associate a white cue with a reward, and a black cue with the absence of a reward. A cue consisted of a bowl (5 × 3 cm, Ø × H), in front of a laminated card (9 cm^2^) of matching colour. To teach the birds to associate the cues with their outcomes, we simultaneously presented them with both a rewarded white cue and an unrewarded black cue, separated by an opaque divider, several consecutive times. To prevent the development of side preferences, we varied which side the rewarded cue was presented on (left or right) according to a pre-determined, pseudorandom sequence. For each trial, there were three possible outcomes: “pass”, in which the bird approached the rewarded cue without needing help, “fail”, in which the bird approached the unrewarded cue or left the arena, or “helped”, in which the bird was helped to find the rewarded cue (note that we only initially helped birds and not during testing). We deemed a bird ready to progress to the testing phase once it had scored six consecutive passes. This criterion was chosen as it is very unlikely to be reached by chance (56). The number of trials birds received in this training stage varied depending on how many they needed to reach our set learning criterion. In terms of sessions, birds typically needed 1 or 2 (max 4) to reach our set learning criterion.

#### Testing Phase

During cognitive judgement bias testing, we presented birds with single cues, either rewarded white, unrewarded black, or one of three novel, unrewarded grey cues (i.e., light grey: 25% white/75% black, mid grey: 50% white/50% black, or dark grey: 75% white/25% black) in a pseudorandom order over a series of trials. Three different grey cues were used due to other investigations which also used data from this test. For this study, we used response to the mid grey cue to measure positive affective state, as this cue is the most ambiguous between the learnt positive and negative cue. Birds saw the mid grey cue 2 times in 2016 and 3 times in 2017 and 2019. To measure “Positive affective state,” we recorded average latency (s) to approach the mid grey cue. A shorter latency indicated higher optimism and, thus, a more positive affective state (9, 33, 34). Note the inverse nature of this measure. We also recorded each bird's average latency to approach the rewarded cue in this test. We gave chicks up to 30 s and adults up to 60 s to approach the cue (since adults were in larger arenas and can show lower food motivation than chicks). Ten younger chicks, eight older chicks and 13 adults were given max values in this test. Seventeen younger chicks, six older chicks, and 29 adults did not complete the cognitive judgement bias test, either because they failed to learn the cue reward association at the training stage or did not complete the test. The latter was due to low food motivation. Variation in our positive affective state measure ranged from 0.93 to 30 s for chicks and 1.70 to 60 s for adults.

### Statistical Analyses

We used R studio version 4.1.2 (67) to analyse our data. As the data did not fit assumptions of normality, we used non-parametric tests. We considered *p*-values ≤ 0.05 to imply significant results.

We explored temporal consistency in our measures of inhibitory control and affective states between younger chicks and adults (in Cohort 1), and temporal consistency in our measure of inhibitory control between younger chicks and older chicks (in Cohort 3), using Spearman's rank correlation tests.

As fowl can display changes in behaviour and cognition, and relationships between these, over ontogeny [e.g., (34, 57–59)], we analysed data from younger chicks, older chicks, and adults separately. To explore how inhibitory control related to affective state in our birds, we created models using the package “lme4” (68). Before designing the models, we investigated how independent our two affective state measures were by exploring the relationship between them, within each age, using Spearman's rank correlation tests. We found that “Negative affective state” (i.e., latency to righten in a tonic immobility test) and “Positive affective state” (i.e., latency to approach a novel, ambiguous cue in cognitive judgement bias test) were not correlated in younger chicks (*R*_s_ = 0.07, *p* = 0.38, *N* = 162) or older chicks (*R*_s_ = −0.22, *p* = 0.11, *N* = 50) and were only moderately correlated in adults (*R*_s_ = −0.29, *p* = 0.01, *N* = 69). Therefore, we made separate models for “Negative affective state” and “Positive affective state” for all ages. Initially, we made a generalised linear mixed model for younger chicks, again using “lme4”, with “Cohort” (1–3) as a random effect. However, as “Cohort” explained very little variation, we used generalised linear models instead, also made in “lme4”. We did not include individual ID as a random effect, since only one measure per age was included in our data. In all our models, our measure of affective states (either “Negative affective state” or “Positive affective state”) was our response variable. For adults and the older chicks, the predictor variables in our models were “Inhibitory control” and an interaction between “Sex” (male = 0, female = 1) and “Inhibitory control.” These predictor variables were also included in the model for younger chicks, along with an interaction between “Cohort” and “Inhibitory control.” We included interactions between “Sex” and “Inhibitory control,” and “Cohort” and “Inhibitory control,” in our models, but did not include “Sex” and “Cohort” as separate predictors. This was because we were specifically interested in whether the relationship between inhibitory control and affective states differed between sex or cohort, not whether affective states differed between sex or cohort. If, for any of our models, an interaction was not significant, we removed this interaction from the model. This resulted in the removal of the interaction between “Sex” and “Inhibitory control” from all our models. The interaction between “Cohort” and “Inhibitory control” was significant in the model for younger chicks, thus we did further analyses to investigate how the relationship between inhibitory control and affective state differed between cohorts. To do this, we first subsetted the data for younger chicks into the three separate cohorts and then ran simple models (affective state measure ~ inhibitory control) for each subset separately. In all our models for “Positive affective state,” we included average latency to approach the rewarded cue as a covariate, thus accounting for individual differences in response speed not due to differences in optimism (e.g., general speed, motivation). For all our models, our response variables were continuous and non-normal, thus we used a gamma distribution. As gamma requires only positive values in response variables, we replaced 0 s in the data with 0.01.

As results can be influenced by outliers or max values (the presence of max values could create ceiling effects that mask relationships in the data), we ran the analyses first with all data, and then (1) with outliers removed (first only extreme outliers and then also mild outliers, defined below), and (2) with max values removed. We defined extreme outliers as data points that were 3 × the interquartile range of the upper or lower quartiles, and mild outliers were as data points that were 1.5 × the interquartile range of the upper or lower quartiles (69, 70). Max values were measures of 600 for “Negative affective state,” 30 for “Positive affective state” in younger and older chicks, 60 for “Positive affective state” in adults.

## Results

We found no qualitative effects of outliers or max values on any of our analyses, that is the patterns we detected, and what we found to be significant or non-significant, did not differ between analyses using all data, analyses using data with outliers removed, or analyses using data with max values removed. Therefore, we here only report results from analyses using all data. We found no sex differences in relationships between inhibitory control and affective states for any of our ages (in all models the interaction between sex and inhibitory control was *p* > 0.1).

Inhibitory control was not consistent between younger chicks and adults (in Cohort 1, *R*_s_ = −0.07, *p* = 0.64, *N* = 42), though it was consistent between younger chicks and older chicks (in Cohort 3, *R*_s_ = 0.32, *p* = 0.02, *N* = 54). Our measure of negative affective state did not show consistency between younger chicks and adults (in Cohort 1, *R*_s_ = 0.006, *p* = 0.97, *N* = 42), nor did our measure of positive affective state (*R*_s_ = −0.13, *p* = 0.51, *N* = 41).

### How Inhibitory Control Linked to Affective States in Younger Chicks

**Figure 1:**
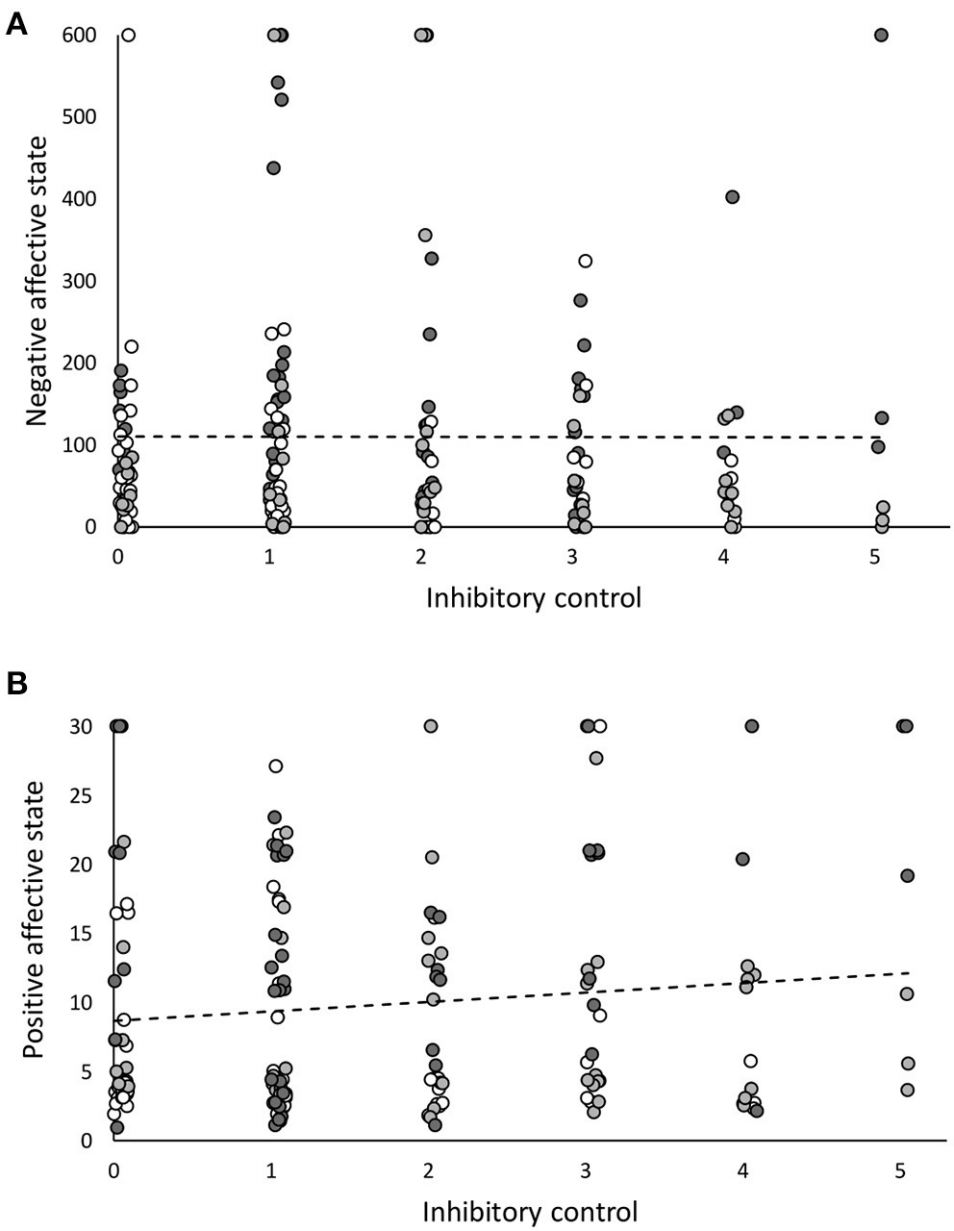
The relationship between inhibitory control and affective state in younger red junglefowl chicks (aged around 2.5 weeks old, *N* = 180). Plots show the weak, yet significant relationships between inhibitory control and **(A)** negative affective state, **(B)** positive affective state. Inhibitory control is the number of trials, out of 5, in a detour task, in which an individual uses a learnt detour to obtain a reward from the centre of a transparent tube (a higher measure indicates better inhibitory control). Negative affective state was measured as latency to return to standing in a tonic immobility test (a higher measure indicates a more negative affective state). Positive affective state was measured as latency to approach an ambiguous cue between a learnt rewarded and a learnt unrewarded cue in a cognitive judgement bias test (a lower measure indicates a more positive affective state, observe the reversed nature of this). Each data point represents an individual bird, and the data are from all cohorts (Cohort 1 = white, Cohort 2 = mid grey, Cohort 3 = dark grey) and both sexes pooled. Dotted lines are trendlines.

In younger chicks (i.e., ≈2.5 weeks old), birds that had higher “Inhibitory control” had a significantly higher “Negative affective state” (*t* = 2.49, estimate = 0.005, SE = 0.002, *p* = 0.01, *N* = 177, Figure 1A). Further, the interaction between “Cohort” and “Inhibitory control” was a significant predictor of “Negative affective state” (*t* = −2.89, estimate = −0.002, SE < 0.001, *p* = 0.004). However, the relationships between “Inhibitory control” and “Negative affective state” between cohorts were all non-significant when cohorts were analysed separately (Cohort 1: *t* = 0.88, estimate < 0.001, SE = 0.002, *p* = 0.88, *N* = 70; Cohort 2: *t* = 0.84, estimate = 0.02, SE = 0.002, *p* = 0.41, *N* = 49; Cohort 3: *t* = −0.37, estimate < 0.001, SE < 0.001, *p* = 0.69, *N* = 58). Younger chicks with better inhibitory control also had a higher “Positive affect state” measure (*t* = 2.75, estimate = 0.04, SE = 0.01, *p* = 0.006, *N* = 164, Figure 1B; recall that a higher “Positive affective state” measure implies a less positive affective state, because the measure used was latency to approach the ambiguous cue). The interaction between “Cohort” and “Inhibitory control” was also a significant predictor of “Positive affective state” (*t* = −3.44; estimate = −0.02, SE = 0.004, *p* < 0.001). When the relationship between “Inhibitory control” and “Positive affective state” was looked at within each cohort separately, it was non-significant in Cohort 1 (*t* = 0.061, estimate = 0.001, SE = 0.016; *p* = 0.95, *N* = 66), Cohort 2 (*t* = 0.91, estimate = 0.007, SE = 0.008, *p* = 0.37, *N* = 46), and Cohort 3 (*t* = −1.19, estimate = −0.04, SE = 0.005, *p* = 0.41, *N* = 52). Thus, in younger chicks, inhibitory control was positively linked to negative affective state and negatively linked to positive affective state.

### How Inhibitory Control Linked to Affective States in Older Chicks

In older chicks (i.e., ≈5 weeks old), “Inhibitory control” did not link to either “Negative affective state” (*t* = −0.55; estimate = < -0.001, SE = 0.004, *p* = 0.59, *N* = 58) or “Positive affective state” (*t* = 0.73; estimate = 0.002, SE = 0.004, *p* = 0.79, *N* = 0.93).

### How Inhibitory Control Linked to Affective States in Adults

In adults (i.e., ≈28 weeks old), “Inhibitory control” did not link to either “Negative affective state” (*t* = −1.62; estimate = -0.001, SE = 0.001, *p* = 0.11, *N* = 96) or “Positive affective state” (*t* = −0.59; estimate = 0.002, SE = 0.003, *p* = 0.56, *N* = 70).

## Discussion

We here investigated whether inhibitory control linked to affective states in red junglefowl at three different ages (younger chicks, older chicks, and adults, thus two different developmental stages, chicks and adults) and in both males and females. Our measures of affective states were not correlated. Regarding relationships between inhibitory control and affective states, we found that, in younger chicks, poorer inhibitory control was linked both to a less negative affective state (significantly, yet weakly) and, somewhat stronger, to a positive affective state (again significantly, though still weak). We found no links between inhibitory control and either positive or negative affective states in older chicks, or adults. Finally, we found no sex differences in relationships between inhibitory control and affective states, at any age.

Our measure of negative affective state did not correlate with our measure of positive affective state. This indicates that our affective state measures are two (relatively) independent measures, which capture separate aspects of affective states. This supports the concept that affective states are not unilinear, that is, that positive affective state cannot simply be considered the opposite of negative affective state, or vice versa (33, 71). To put it another way, optimism is not simply a lack of fearfulness and fearfulness is not simply a lack of optimism. Thus, by using multiple tests of affective state here, we could get at both positive and negative affective states and so explore the effects of inhibitory control on affective states at a broader level, than if we had only focused on one aspect of affective state. We recommend future researchers to do likewise. An interesting avenue for future research could be to explore a more complete description of the aspects of affective state in animals.

Our measures of inhibitory control, negative affective state, and positive affective state, were not consistent over the transition from chickhood to adulthood. On the other hand, inhibitory control was found to be moderately consistent over a shorter time span (between younger and older chicks). That measures were not consistent between chicks and adults is not unexpected, as red junglefowl undergo two major developmental changes, during this transition, which could potentially result in changes to their behaviour and/or cognition. First, becoming fully independent from their mother [at around 10–12 weeks of age, (60, 61)] and second, sexual maturation [at around 24–25 weeks of age, (62, 63)]. That our measure of inhibitory control was consistent over a shorter time span suggests that this captures individual variation, at least to some extent. We could not here explore temporal consistency in negative, or positive affective state, over shorter time spans, as we only took one measure of these per developmental stage (i.e., as chicks or as adults). Previous studies, on this species, have found negative affective state to be moderately consistent over time, within chicks (58) and adults (57), and positive affective state to be weakly consistent over time (and mainly driven by environmental effects), within chicks (34, 56). We acknowledge that, as temporal consistency in our measures varied (from low to moderate), and we used single, rather than repeated, measures of behavioural variation in our analyses, we here used unpartitioned phenotypic correlations to investigate among-individual patterns. This approach, though common practise in animal behaviour research, is problematic. While behavioural correlations may reflect among individual correlations, this should not be assumed outright (72, 73). Thus, further research able to partition variation of within and between individual patterns is needed to determine whether the patterns we observed here, reflect patterns found on the between individual level.

For the current work, we made two hypotheses. First, if inhibitory control linked to affective states via risk seeking vs. risk avoidance, and individuals with a poorer inhibitory control would be expected to have less negative, more positive, affective states. Second, if inhibitory control linked to affective states due to poorer inhibitory control having negative consequences, individuals with poorer inhibitory control were predicted to have more negative, less positive, affective states. Our results for younger chicks offer support for our first hypothesis, and not for our second hypothesis, in that we found a link between poorer inhibitory control and less negative, more positive, affective states. Thus, in younger chicks, poorer inhibitory control appeared to be associated with better welfare, if welfare is indicated by more positive, less negative affective states. In older chicks and adults, we found no connections between inhibitory control and affective states. Our second hypothesis was partly based on previous findings that individuals with poorer inhibitory control are less able to adapt to changing situations. However, our birds were not exposed to many changing situations in which being less able to adapt could have been expected to influence affective states. This is based on that (besides from Cohort 2, i.e., younger chicks tested in 2017), our birds did not experience changing situations in terms of where they were housed, or who they were housed with. Further, all birds were carefully habituated to the main change of situation they regularly experienced (i.e., taking part in testing) to reduce this causing stress. We also based our second hypothesis on previous findings that individuals with poorer inhibitory control are more likely to display behaviours that can have negative connotations [e.g., (25–27)], and that stress can worsen inhibitory control (28, 29). Our methods may have prevented such behaviour and stress from influencing affective states in our birds. First, we did not observe birds behaving in ways similar to described in previous work [e.g., feather pecking, vent pecking (26, 27, 49)]. Moreover, we tried to avoid stressing our birds as much as possible. Studies are needed to further explore and directly tests potential relationships between inhibitory control and affective states. This can be done, for example, by manipulating affective states (e.g., through providing enrichment, or stressors) and measuring how this affects inhibitory control. Such studies are currently scarce. So far, exposure to enrichment or stressors seem to result in poorer inhibitory control (28, 29, 40, 74, 75), though more research is needed to test the generality of this. As well as exploring how affective states directly affect inhibitory control, we encourage studies which aim to better understand how inhibitory control links to known welfare issues, for example, feather pecking. Feather pecking is a major welfare issue for chickens (27), which are one of the world's most intensively farmed animals [reviewed by (47, 48)]. The relationship between feather pecking and inhibitory control is currently unclear as, while the idea that feather pecking results from higher impulsivity has some support [e.g., (26, 27)], this is not always the case [e.g., (76)]. More research is therefore needed to disentangle the relationship between feather pecking and inhibitory control, as well as to explore how inhibitory control relates to other welfare issues.

We here assessed negative affective state with a tonic immobility test and positive affective state with a cognitive judgement bias test. While these tests are both well-established (7, 77) and can be used in a variety of species (7, 54, 55, 77, 78), they have their potential drawbacks. The reaction seen in tonic immobility test is thought to have developed as a defensive reaction to a predator attack (79, 80). Based on this, the tonic immobility test could be assumed to mimic a predator attack and, consequently, to be stressful and/or fear inducing to animals that experience it. Thus, the tonic immobility test itself could have negative implications for welfare if used frequently. The cognitive judgement bias test, while not intrinsically stressful, can be costly in terms of time, as animals need to be trained to reach a learning criterion before they can be tested. There are, therefore, incentives to develop less stressful and simpler ways to assess affective states. Especially useful would be single tests that can assess both positive and negative affective states simultaneously. That, in younger chicks, measures from the detour task associated with measures from both the tonic immobility test and the cognitive judgement bias test could imply that a detour task could, in some cases, function as such a test. However, we would advise against this. First, the relationships we found between inhibitory control and affective states were weak. Furthermore, various factors, besides inhibitory control and affective states, may affect an individual's performance in a detour task. These include variation in food motivation, differences in how individuals are trained for the detour (81), and learning ability [as individuals can learn to improve inhibitory control over time (18, 40)]. To reduce the influence of these factors, we used a reward with high motivation for both chicks and adults. Further, we ensured that all birds were taught the detour in the same way and the number of trials birds had in this test was kept low (to avoid effects of learning). Another controversial aspect of the detour task is that its results do not necessarily correlate with other measures of inhibitory control [e.g., (82, 83)]. This, however, does not necessarily imply that detour tasks do not measure inhibitory control, but rather that inhibitory control is a complex construct consisting of distinct aspects (82, 83), one of which the detour task captures. Nevertheless, we acknowledge that we here explored the relationship between a particular aspect of inhibitory control and affective states, and that, to determine relationships between inhibitory control and affective states, in general, a battery of inhibitory control tasks should perhaps be used, to capture a broader picture of inhibitory control.

By collecting data from younger chicks, older chicks, and adults we were able to explore whether relationships between inhibitory control and affective states differed over ontogeny. Our results suggested that they were (since patterns were detected in younger chicks, but not in older chicks or adults). We acknowledge that we may have found links between inhibitory control and affective states in younger chicks only, because our sample size for younger chicks was larger than our sample size for older chicks and adults. However, finding differences over ontogeny in relationships between inhibitory control and affective states in red junglefowl, could be expected. Fowl are known to differ as they age in other aspects of behaviour and cognitive performance, including relationships between these [e.g., (57–59)]. These changes in behaviour and cognitive performance may be due to social and physiological changes which occur during maturation from chicks to adults [reviewed in (57)]. However, how, and why, social and physiological changes may affect behaviour or cognition is not fully understood. That younger chicks which were more impulsive in a detour task were also showed more fearful responses in a tonic immobility test and were less optimistic in a cognitive judgement bias test indicates that these chicks may have had a proactive-reactive behavioural syndrome. More proactive individuals are typically less fearful, more impulsive, and more optimistic, than more reactive individuals (84–87). That measures of impulsivity, fearfulness and optimism did not correlate in older chicks or adults suggests that this syndrome may fade as birds age. Another reason why relationships between inhibitory control and affective states change over ontogeny could be because individuals, including red junglefowl, can improve their inhibitory control over time (18, 40). Overall, our results suggest that age can affect the relationship between inhibitory control and affective states (both negative and positive), and we encourage future research to investigate the generality of this further. This could be done, for example, by measuring how individuals' inhibitory control relates to affective states at multiple points during their lives.

Previous work, in other species, has shown that both inhibitory control and affective states can differ between sexes (43, 44). Therefore, the relationship between inhibitory control and affective states could also be expected to differ between sexes, especially species which can show sex differences in behaviour, such as fowl (57, 88). Nevertheless, we did not observe sex differences in relationships between inhibitory control and affective states in the red junglefowl used in this study. Earlier studies have also found a lack of, or only weak, sex differences in aspects of behaviour [e.g., tonic immobility (57)] or cognition [e.g., learning speed in discrimination and spatial learning tests (64)] in this species. Regardless, we still encourage future studies on relationships between cognition and affective states to investigate sex differences in these relationships where possible.

Overall, we here show that, based on behavioural correlations, inhibitory control seems to link to both positive and negative affective states, and thus in turn, that inhibitory control, an aspect of cognition, may have implications for welfare. However, the nature of links we observed between inhibitory control and affective states varied over ontogeny. Such links may also differ between sexes, though we found no evidence of that here. Overall, this study, along with other recent studies [e.g., (11, 12)], suggests that individual variation in cognition can link to affective state, knowledge which in turn could help us to improve the welfare of animals [e.g., (89–91)].

## Data Availability Statement

The original contributions presented in the study are included in the article/Supplementary Material, further inquiries can be directed to the corresponding author.

## Ethics Statement

The animal study was reviewed and approved by Linköping Ethical Committee, Linköping District Court (ethical permit numbers 50-13 and 288-2019).

## Author Contributions

LG came up with the idea for the study. LG and HL designed the study. LG collected data together with members of the Løvlie Group (see acknowledgements) and analysed it based on communication with HL. LG wrote the manuscript with input from HL and CC. All authors contributed to the article and approved the submitted version.

## Funding

HL received funding from the Swedish research council FORMAS (Grant No. 2015-11891) and LiU Neuro Systemsbiology for costs associated with housing of birds or paying of PhD-salary (to LG).

## Conflict of Interest

The authors declare that the research was conducted in the absence of any commercial or financial relationships that could be construed as a potential conflict of interest.

## Publisher's Note

All claims expressed in this article are solely those of the authors and do not necessarily represent those of their affiliated organizations, or those of the publisher, the editors and the reviewers. Any product that may be evaluated in this article, or claim that may be made by its manufacturer, is not guaranteed or endorsed by the publisher.
